# Intracochlear electrode array position and cochlear implant outcomes using the nucleus slim modiolar electrode and the extended round window approach: a follow-up study

**DOI:** 10.1007/s00405-021-07247-w

**Published:** 2022-01-18

**Authors:** Tim M. Klabbers, Floris Heutink, Wendy J. Huinck, Willem-Jan van der Woude, Berit M. Verbist, Emmanuel A. M. Mylanus

**Affiliations:** 1 Department of Otorhinolaryngology and Head and Neck Surgery, Radboud University Medical Center, Philips Van Leydenlaan 15, 6525 EX Nijmegen, The Netherlands; 2 Department of Radiology, Radboud University Medical Center, Nijmegen, The Netherlands; 3 Donders Institute for Brain, Cognition and Behaviour, Nijmegen, The Netherlands; 4 Department of Radiology, Leiden University Medical Center, Leiden, The Netherlands

**Keywords:** Cochlear implantation, Residual hearing, Slim modiolar electrode, Extended round window approach

## Abstract

**Purpose:**

The aim of this study was to evaluate the intracochlear position of the Slim Modiolar Electrode (SME) after insertion via the extended Round Window (eRW) approach, and to correlate this with residual hearing preservation and speech perception outcomes.

**Methods:**

Twenty-three adult participants, consecutively implanted with the SME via the eRW approach, were included in this prospective, single-center, observational study. Electrode position was evaluated intra-operatively using X-ray fluoroscopy and TIM measurement, and post-operatively using ultra-high resolution CT. Residual hearing [threshold shift in PTA between pre- and post-operative measurement, relative hearing preservation (RHP%)] and speech perception were evaluated at 2 and 12 months after surgery.

**Results:**

In each of the 23 participants, complete scala tympani positioning of the electrode array could be achieved. In one participant, an initial tip fold-over was corrected during surgery. Average age at implantation was 63.3 years (SD 13.3, range 28–76) and mean preoperative residual hearing was 81.5 dB. The average post-operative PTA threshold shift was 16.2 dB (SD 10.8) at 2 months post-operatively, corresponding with a RHP% score of 44% (SD 34.9). At 12 months, the average RHP% score decreased to 37%. Postoperative phoneme scores improved from 27.1% preoperatively, to 72.1% and 82.1% at 2 and 12 months after surgery, respectively.

**Conclusion:**

Use of the eRW approach results in an increased likelihood of complete scala tympani insertion when inserting the SME, with subsequent excellent levels of speech perception. However, residual hearing preservation was found to be moderate, possibly as a result of the extended round window approach, emphasizing that it is not an all-purpose approach for inserting this particular electrode array.

## Introduction

With the expansion of the indication criteria for cochlear implantation (CI), preservation of residual hearing has become an important objective [1]. An increasing body of evidence suggests that preservation of residual hearing and overall audiological outcomes are directly influenced by the occurrence of insertional trauma during cochlear implantation [2]. Mechanical trauma during electrode insertion can include osseous spiral lamina fracture, lateral wall or modiolar injury, basilar membrane disruption and electrode translocation from the scala tympani (ST) to the scala vestibuli (SV) [3]. In some cases, the electrode is even inserted directly into the SV due to misplacement of the cochleostomy [4]. Several studies conclude that complete placement of the active electrode within the ST is associated with better performance with the CI, compared to partial or complete SV placement [5–7]. This has led to an increased interest in the surgical approach to the cochlea, hypo-traumatic surgical techniques and the development of hypo-traumatic electrode arrays.

Currently available CI electrode arrays can be categorized into two main types: ‘straight’ or ‘pre-curved’. Straight electrode arrays essentially follow the lateral wall of the cochlea once implanted. Pre-curved, or (peri)modiolar electrode arrays on the other hand, are designed to curl around the medial wall and position the electrode contacts in proximity to the modiolus and spiral ganglion cells, thus leading to a reduced spread of excitation, lower stimulation thresholds and improved stimulation specificity [8–11]. However, conventional perimodiolar electrodes are known to lead to a higher rate of translocation to the scala vestibuli, resulting in poorer speech perception outcomes [12]. Advancements in the design of pre-curved electrode arrays led to the development of the slim modiolar electrode (SME) in 2016; an electrode that is 60% thinner and more flexible than the previous generation of perimodiolar electrodes produced by the same manufacturer [13]. This electrode is designed to minimize trauma during insertion and to preserve residual hearing.

However, a recent study by Heutink et al. in 23 adult participants implanted with a SME via the cochleostomy (CS) approach revealed a translocation from the scala tympani to the scala vestibuli in 36.4% of the participants [4]. Translocation was subsequently observed to lead to significantly lower levels of residual hearing preservation (RHP) (19.7% compared to 77.2% in participants with full ST placement) and lower speech recognition scores.

Several studies, therefore, advocate the use of either the round window (RW) or extended round window (eRW) insertion approach when using the SME, as this has been shown to decrease the probability of scalar excursion and thus improve audiological outcomes and hearing preservation [2, 12, 14]. These two approaches are, however, often grouped together and studies solely investigating outcomes of implantations of the SME via the extended round window approach are scarce. The primary objective of this prospective study was to evaluate the intracochlear position (i.e. occurrence of translocation from scala tympani to scala vestibuli, presence of a tip fold-over) of the SME after implantation using the extended round window technique and to correlate this to post-operative audiological outcomes such as hearing preservation and speech perception. Additionally, we aim to compare these findings to previously published results obtained with the SME using different surgical approaches.

## Methods

### Study design

This was a prospective, single-center observational study in twenty-three adult patients consecutively implanted with the Slim Modiolar Electrode (SME; Nucleus® CI532/CI632; Cochlear Ltd., Sydney, Australia) using the extended Round Window (eRW) approach. Participants were included between July 2019 and July 2020. Exclusion criteria were (1) pre-lingual onset of deafness, (2) anatomical variations of the cochlea possibly influencing electrode insertion and (3) inability to undergo CT-scanning. Preoperatively, demographic data including the history of hearing loss and audiometry were collected. X-ray fluoroscopy and trans-impedance matrix (TIM) measurements were performed intra-operatively to rule out tip fold-overs. Electrode position was evaluated 1 week post-implantation using an ultra-high resolution computed tomography (UHRCT) scan. Residual hearing was evaluated routinely 2 months post-operatively, whilst speech perception was measured routinely 2 and 12 months post-operatively. Several participants included in the current study were also reported on in a proof-of-concept article by Klabbers et al. on the use of TIM measurements for initial tip fold-over detection. However, no clinical outcomes were evaluated in the previous study. All procedures performed in this study were in accordance with the 1964 Helsinki Declaration and its later amendments [15]. Institutional Review Board approval was obtained and all patients signed informed consent forms. The study was registered in the Netherlands Trial Register (trial number NL8290).

### Surgical procedure

**Fig. 1:**
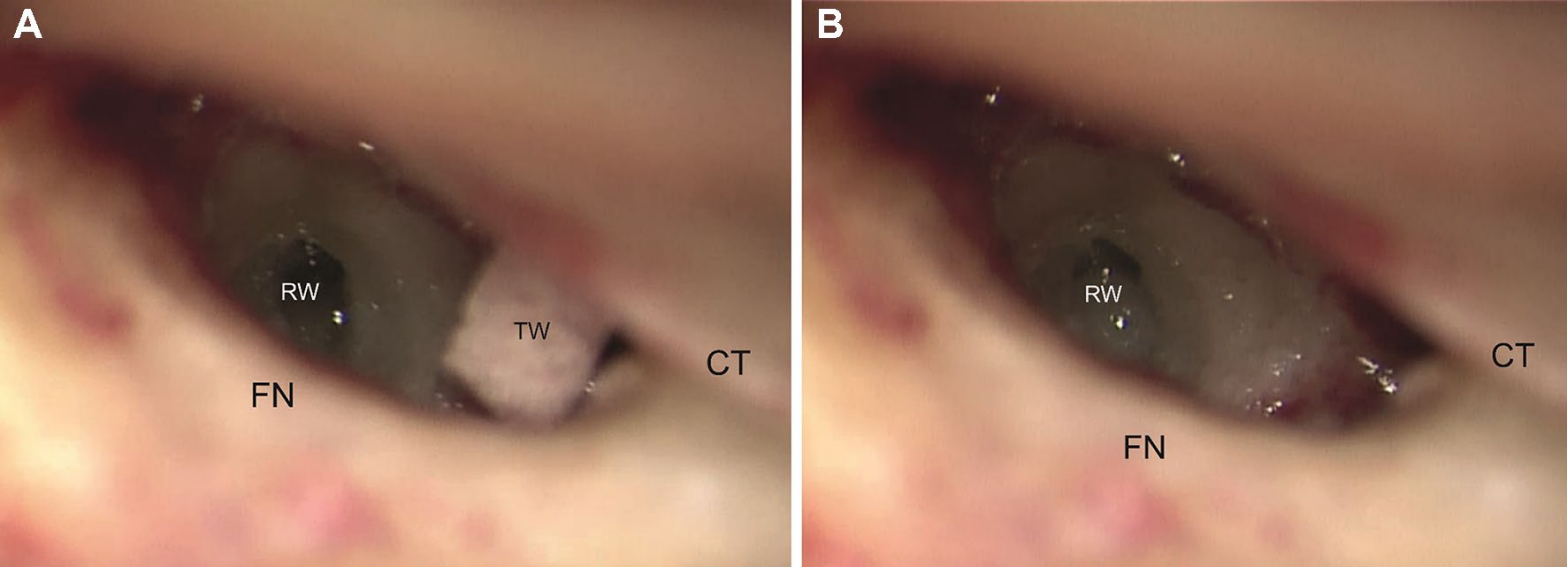
Intraoperative view of the round window of a right ear through the facial recess after completion of the cortical mastoidectomy and posterior tympanotomy. **A** Before antero-inferior drilling and **B** after antero-inferior drilling. *FN* facial nerve, *RW* round window, *TW* tissue wipe, *CT* chorda tympani nerve

Surgery was performed by three experienced CI-surgeons, and the standard mastoidectomy and facial recess approach was used to gain access to the cochlea in all cases. The bony overhang of the round window niche was carefully removed and the scala tympani was opened using the extended round window technique, in which a so-called round window margin cochleostomy is created by carefully drilling the anterior–inferior bony margin and crista fenestrae (Fig. 1). All 23 patients were implanted with the pre-curved, slim modiolar electrode (SME). The SME has an active component with a length of 14 mm and a diameter of 0.35 mm × 0.4 mm apically and 0.45 mm × 0.5 mm basally. It is loaded into a protective plastic sheath of 5 mm in length and 0.77 mm in diameter and is inserted into the cochlea until the sheath stopper reaches the round window opening. Using forceps, the SME array is then slowly advanced until fully inserted (approx.120 s), after which the sheath is carefully retracted and removed. Depending on the surgeons’ preferences, the extended round window opening was sealed with periosteum or fascia and fibrin glue. During surgery, patients were given a single dose of 1.8 mg/kg intravenous methylprednisolone.

### Evaluation of intracochlear electrode position

**Fig. 2:**
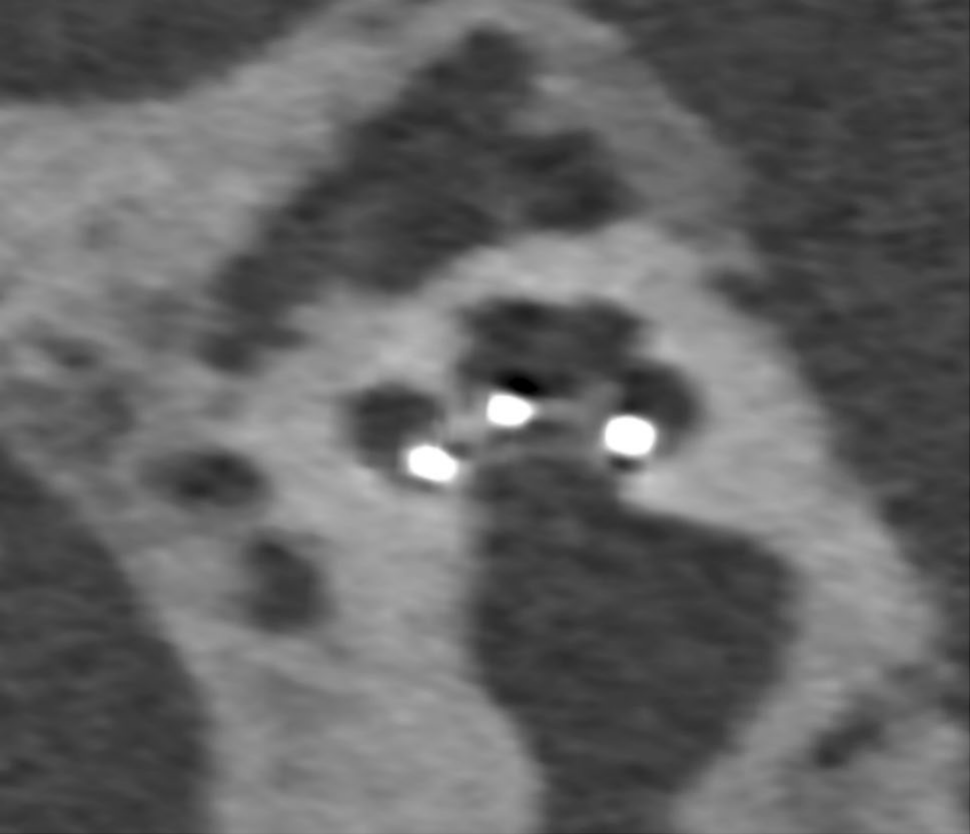
Mid-modiolar section of the post-implantation UHRCT of participant #12 indicating scala tympani placement of the electrode array

Directly following electrode insertion and packing, TIM measurements and fluoroscopy were performed to rule out electrode array tip fold-over. In the event of a tip fold-over, the array was retracted and repositioned in the same surgical setting. One week post-operatively, electrode array position was evaluated using a UHRCT-scan (Aquilion Precision, Canon Medical Systems, Otawara, Japan). For scalar position analysis, the UHRCT-images were reconstructed using multiplanar reconstruction (MPR) and filtered back projection (bone filter kernel FC81), with a field of view of 90 mm, 1024 × 1024 matrix and 0.25 mm slice thickness. Mid-modiolar sections of the reconstructed UHRCT-scan images were evaluated by an experienced head and neck radiologist (Fig. 2). Each of the twenty-two electrode contacts was individually assessed and scored as either located in the ST or SV.

### Audiological evaluation

Unaided pure tone audiometry at 125, 250, 500, 1000 and 2000 Hz was performed pre- and post-operatively for both ears using headphones in a soundproof booth following standard audiometry and masking protocol at our center. The low frequency pure tone average (LFPTA), defined as the average threshold at 250, 500 and 1000 Hz, was calculated for each patient. If a patient failed to respond to the stimulus at a certain frequency, the maximum stimulation level (MSL) was recorded (as per Skarzynski et al. [16]). For the frequencies evaluated in this study, MSLs were 90 dB, 105 dB, 110 dB, 120 dB and 120 dB, respectively. Failure to respond to two or more of the frequencies for calculating the LFPTA was recorded as “non-measurable hearing” (NMH). In accordance with the consensus paper by the HEARRING group, residual hearing preservation (RHP) was calculated using the hearing preservation classification score; $RHP\% = \left[ {1 - \left( {\frac{{LFPTA_{post} - LFPTA_{pre} }}{{LFPTA_{max} - LFPTA_{pre} }}} \right) \times 100\% } \right],$ where LFPTA_max_ is defined as the average of MSLs at 250, 500 and 1000 Hz [16].

RHP percentages for each participant were categorized as either (1) complete preservation (> 75%), (2) partial preservation (25–75%) or (3) minimal preservation (0–25%). Additionally, mean threshold shifts in LFPTA between pre- and post-operative testing were evaluated. If necessary, these values were corrected for the natural progression of hearing loss, as previously described by Snels et al. ($\mathrm{\Delta LFPTA CI ear }-\mathrm{ \Delta LFPTA contralateral }(\mathrm{CL})\mathrm{ ear}$) [17].

Speech perception in quiet was evaluated routinely at 2 and 12 months post-operatively. The standard test to assess speech perception in the Netherlands is issued by the Dutch Society of Audiology (NVA) and consists of a list of phonetically balanced, monosyllabic consonant–vowel-consonant (CVC) words presented at 65 dB SPL through a speaker which is placed in front of the participant in a soundproofed booth. The average percentage of three CVC lists was calculated.

### Statistical analysis

Statistical evaluation was performed using SPSS (version 25, Armonk, New York). Continuous data are reported as mean values with standard deviations and comparisons between the audiological outcomes of the current and previous study were performed using independent samples *T* tests. *P* values < 0.05 were deemed statistically significant.

## Results

### Demographics

Twenty-three patients were consecutively implanted with the SME at our center. Of these patients, 11 (47.8%) were male and 12 (52.2%) female. Fourteen patients (60.9%) were implanted in the right ear, whilst nine (39.1%) were implanted in the left ear. The average age at implantation was 63.3 years (SD 13.3; range 28–76) and participants had a mean preoperative residual hearing (LFPTA) of 81.5 dB (SD 11.9).

### Scalar position and tip fold-over evaluation

Upon post-operative UHRCT scan evaluation, all of the electrode contacts for each of the 23 patients included in this study were found to be located fully within the scala tympani: no translocation had occurred. Intraoperative fluoroscopy and TIM measurement revealed a tip fold-over in 1 of the 23 patients (4.3%). In this patient, subsequent repositioning of the electrode array in the same surgical setting led to a satisfactory intracochlear position as confirmed by repeated fluoroscopy and TIM measurements, as well as a complete ST positioning of the array as confirmed with UHRCT 1 week post-operatively. Postoperative UHRCT evaluation did not reveal a tip fold-over in any of the other participants.

### Audiometry

Postoperative residual hearing thresholds and speech perception scores for all twenty-three participants are presented in Table 1. The mean threshold shift in LFPTA at activation across all participants without tip fold-over was 16.2 dB (SD 10.8), whilst the mean RHP% was 43.9% (SD 34.8) (Table 2). The mean threshold shift in LFPTA in the contralateral, non-implanted ear was 0.1 dB and, therefore, no correction for the natural progression of hearing loss was deemed necessary.

**Table 1:** Audiometric data

ID	Gender	Age	Etiology	CI side	Tip fold-over	Scalar location	LFPTA (in dB)				2-month RHP		12-month RHP		Postoperative speech recognition (CVC-phoneme scores)		
							preLFPTA CI ear (dB)	preLFPTA CL ear (dB)	Δ LFPTA^a^ CI ear (dB)	Δ LFPTA CL ear (dB)	%	Cat	%	Cat	Pre-operative	2 months	12 months
1^b^	M	28	Mitochondropathy	R	N	ST	66.7	CI	45	CI	0%	M	0%	M	56	59	56
2	F	60	Hereditary	R	N	ST	70	70	5	– 5	88%	C	60%	P	39	90	87
3	M	70	Usher IIa	R	N	ST	98.3	98.3	13.4	– 6.7	0%	M	0%	M	18	48	79
4	F	70	Unknown	R	N	ST	80	56.7	18.3	0	42%	P	–	–	24	73	87
5	F	61	Unknown	R	N	ST	75	88.3	8,3	– 3.3	77%	C	77%	C	27	50	66
6	F	65	Unknown	L	N	ST	90	48.3	6.7	6.7	69%	P	53%	P	-	69	90
7	F	69	DFNA9	L	N	ST	85	83.3	21.6	–	19%	M	6%	M	57	92	86
8	M	69	DFNA9	L	N	ST	80	73.3	13.3	0	58%	P	–	–	24	63	82
9	M	71	DFNA9	L	N	ST	76.7	78.3	21.6	6.7	38%	P	5%	M	27	80	83
10	M	65	Unknown	R	Y ^c^	ST	101.7	90	10	13.3	0%	M	–	–	24	93	93
11	M	70	DFNA9	R	N	ST	88.3	75	23.4	0	0%	M	15%	M	15	74	84
12	F	73	Meniere	R	N	ST	88.3	NMH	23.4	–	0%	M	0%	M	0	79	72
13	M	73	Unknown	L	N	ST	85	85	21.67	3.33	19%	M	38%	P	12	84	82
14	F	67	Hereditary	L	N	ST	81.7	70	3.3	1.6	89%	C	78%	C	38	-	59
15	F	69	Unknown	R	N	ST	91.7	81.7	18.3	– 8.4	9%	M	17%	M	12	89	84
16	F	67	DFNA9	R	N	ST	81.7	56.7	28.3	1.6	6%	M	0%	M	10	89	95
17	M	76	Unknown	R	N	ST	76.7	68.3	10	11.7	71%	P	67%	P	57	60	68
18	M	74	Hereditary	R	N	ST	80	60	10	– 1.7	68%	P	84%	C	15	-	96
19	F	70	Unknown	L	N	ST	61.7	58.3	8.3	0	83%	C	80%	C	36	93	86
20	F	65	DFNA9	L	N	ST	53.3	110	35	–	40%	P	57%	P	51	67	84
21	M	49	DFNA9	R	N	ST	98.3	95	1.7	– 1.7	87%	C	–	–	24	66	-
22	F	71	Unknown	R	N	ST	93.3	78.3	16.7	– 3.3	9%	M	20%	M	3	57	86
23	M	80	Unknown	L	N	ST	71.7	55	5	0	88%	C	54%	P	27	26	55
						Mean:	81.5	75.3	16.2	0.09	44%		37%		27.1	72.1	81.1

*LFPTA *low frequency pure tone average (average threshold over 250, 500 and 1000 Hz), *CL*  contralateral, *dB*  decibel, *RHP*   relative hearing preservation (defined by Skarzynski et al. [16] using the following formula: ${\text{RHP}}\, = \,{1}00\, \times \,\left( {{1}\, - \,\Delta {\text{ LFPTA/}}\left( {{\text{LFPTAmax}}{-}{\text{preLFPTA}}} \right)} \right),$ *NMH*  non-measurable hearing (defined as a participant without response at maximum stimulation level at two or more frequencies)

^a^Δ LFPTA = Threshold shift between pre- and post-operative LFPTA (at activation, 2 months post-operatively)

^b^Participant 1 was not included in the post-operative speech perception outcome evaluations due to the retro-cochlear etiology of hearing loss

^c^In participant 10, an intraoperative tip fold-over occurred and, therefore, the residual hearing data were not included in the average outcomes

**Table 2:** Residual hearing outcomes obtained in the current study compared to the results obtained in the previous study by Heutink et al. (CS), as well as specifically for participants in whom the electrode array located in the scala tympani (ST) and those with a translocated electrode array [4]

	eRW (current study)		CS (Heutink et al.)			CS–ST (Heutink et al.)			CS–Translocation (Heutink et al.)		
	Mean (SD)	*N*	Mean (SD)	*N*	*p* value	Mean (SD)	*N*	*p* value	Mean (SD)	*N*	*p* value
Preoperative LFPTA^1^ in dB	81.5 (11.9)	23	92.2 (13.3)	19	*0.009*	94.9 (10.7)	12	*0.003*	87.1 (19)	7	0.296
Postoperative threshold shift in LFPTA in dB	16.2 (10.8)	22	9.2 (9.2)	17	*0.039*	4.4 (5.3)	11	*0.002*	17.9 (9)	6	0.723
RHP% as per Skarzynski^2^	43.9 (34.8)	22	56.9 (46.3)	17	0.269	77.2 (45)	11	*0.022*	19.8 (16)	6	*0.042*

^a^One `participant (#10) was excluded from hearing outcome analysis due to a tip fold-over

^1^Low frequency pure tone average: average threshold for 250, 500 and 1000 Hz

^2^Residual hearing preservation,${\text{RHP}}\% = \left[ {1 - \left( {\frac{{{\text{LFPTA}}_{{{\text{post}}}} - {\text{LFPTA}}_{{{\text{pre}}}} }}{{{\text{LFPTA}}_{{{\text{max}}}} - {\text{LFPTA}}_{{{\text{pre}}}} }}} \right) \times 100\% } \right]$

According to the HEARRING classification, 7 out of 22 (31.8%) participants had complete hearing preservation (RHP > 75%) at activation, whilst 6 out of 22 (27.3%) had partial preservation (RHP 25–75%). 9 out of 22 participants (40.9%) had minimal or no preservation of residual hearing (RHP < 25%). Of the 7 participants who had a preoperative LFPTA of less than 80 dB, only 3 maintained a LFPTA lower than 80 dB at activation.

Long-term residual hearing preservation (RHP%) was evaluated at 12 months post-operatively and was found to have deteriorated by an average of 7% (from 44 to 37%), indicating a progression of residual hearing loss over time.

The mean post-operative CVC-phoneme score at 2 months was 72.1%, and this further improved to 81.1% at 12 months (Table 3).

**Table 3:** Mean speech perception scores obtained in the current study compared to participants with late onset of hearing loss from the previous study by Heutink et al. (CS), as well as specifically for participants in whom the electrode array located in the scala tympani (CS–ST) and scala vestibuli (CS–SV) [4]

	eRW (Current study)		CS (Heutink et al.)			CS–ST (Heutink et al.)			CS–Translocation (Heutink et al.)		
	Mean (SD)	*N*	Mean (SD)	*N*	*p* value	Mean (SD)	*N*	*p* value	Mean (SD)	*N*	*p* value
Preoperative CVC-phoneme score (%)	27.1 (16.9)	22^a^	7.2 (12.9)	12	*0.001*	10.5 (16.5)	6	0.042	3.83 (8.0)	6	*0.003*
Postoperative CVC-phoneme score (%) (2-month)	72.1 (18.0)	21^b^	60.6 (23.4)	11	0.138	72.4 (14.3)	5	0.973	50.83 (26.0)	6	*0.031*
Postoperative CVC-phoneme score (%) (12-month)	81.1 (11.1)	21	77.6 (15.1)	8	0.496	88.3 (10.8)	4	0.251	67.0 (10.9)	4	*0.028*

^a^Preoperative CVC scores were missing for one participant (#6)

^b^One participant (#1) was excluded from post-operative speech perception outcome analysis due to a retro-cochlear etiology of hearing loss and one participant was lost to follow-up (#21)

The post-operative threshold shift in LFPTA in the participant with an intraoperative tip fold-over was 10 dB, whilst the relative hearing preservation score according to the HEARRING classification was 0%. Speech perception scores at 2 and 12 months were 93% and 93%, respectively.

## Discussion

This study was initiated to evaluate the intracochlear electrode position and audiological outcomes of patients with the slim modiolar electrode, implanted exclusively using the extended round window approach. This is currently the approach of choice at our tertiary referral hospital for inserting this particular electrode, as a previous study (conducted at our center) revealed translocations of the SME from scala tympani to scala vestibuli in 36% of the patients implanted via cochleostomy. The electrode arrays of all participants included in the current study were found to be located fully within the scala tympani following extended round window insertion, resulting in excellent levels of speech perception scores.

Implantation of the SME via the extended round window approach, therefore, seems to invariably lead to correct positioning within the scala tympani. These results are an evident improvement compared to previously reported implantations via a cochleostomy approach at our center, and generally comparable to what is reported in the literature, although studies adequately reporting on the CT-evaluated scalar position by surgical approach for the SME are still relatively scarce. The studies in which this was determined using high-resolution imaging result in a combined average translocation risk of 4.8% (22/461) [4, 18–24]. Of these aggregated 461 participants, 396 could be identified as having been implanted using either a RW or eRW approach and 39 as implanted using a CS approach [4, 18, 20, 21, 23, 24]. The translocation risk, extrapolated from these studies, was 3% (12/396) for the eRW/RW approach and 23% (9/39) for the CS approach. Examination of the translocation rates in the studies that additionally discerned between eRW and RW approaches revealed a slightly lower average translocation rate for eRW insertions (0.67%, 1/150 vs. 3.5%, 6/170, respectively) [18, 20, 21, 23]. Therefore, with regards to achieving an optimal scalar position, round window insertion seems the best choice for SME-implantations. Furthermore, extending the round window opening by drilling away the crista fenestrae may result in an even better orientation of the sheath during insertion, with a more direct trajectory down the basal turn and subsequent lower translocation rate [23].

Moreover, our results further confirm that this in turn gives rise to excellent levels of speech perception with this particular electrode. We found the CVC-phoneme score to improve from 27.1% preoperatively to 72.1% at 2 months and 82.1% at 12 months post-operatively. These scores are comparable to, if not better than, what is reported in other studies [19, 21, 23, 25]. Shaul et al. studied 18 patients with similar levels of preoperative phoneme scores implanted with a SME using the eRW approach [26]. They reported an improvement in phoneme scores from 26.1% preoperatively to 73.1% at 3 months and eventually 79.5% at 12 months post-operatively. Unfortunately, most other studies do not report on approach-specific speech perception scores. With regards to implantations via cochleostomy, results of our previous study showed that patients in whom the electrode was found to be located fully within the scala tympani, reached excellent levels of speech perception (improvement from 10.5% preoperatively, to 72.4% and 88.3% at 2 and 12 months post-operatively, respectively), although the number of patients in this group was limited (Table 2). Based on our results, it seems that the eRW approach is the method of choice to achieve consistent scala tympani positioning of the electrode array and excellent levels of speech perception.

However, the residual hearing preservation results obtained in this study using the eRW approach are suboptimal. We found an average loss of 16.2 dB at implant activation, corresponding to a RHP% of 43.9%. These values approximate the results found for patients with cochleostomy-related translocations in whom all contacts were located within the scala vestibuli (17.9 dB and 19.7%, respectively), although again, the number of patients in that study was limited (*n* = 6) [4]. Ramos et al. also showed hearing preservation to be least likely in patients implanted via an extended round window approach, compared to pure round window or cochleostomy approaches, using different electrode types [22]. The mechanism of trauma causing the residual hearing loss, however, must be different from that of insertions via cochleostomy because we did not register any translocations to the SV using the eRW approach. There are several other possible explanations for the lower hearing preservation rates observed using the extended round window approach. One possible explanation is that, although both cochleostomy and extended round window approaches require minimal drilling to create an opening in the cochlea, the insertion directly through the round window additionally results in an increased stiffness of the round window membrane over time. Elliot et al. modelled an estimated 100-fold increase in RW membrane stiffness after cochlear implantation, which was predicted to lead to 20 dB hearing loss across frequencies below 1000 Hz [27]. Furthermore, a study by Rowe et al. investigating residual hearing loss for different routes of electrode insertion using a Guinea pig model showed a pronounced delayed, low frequency hearing loss in round window insertions, which was not observed after cochleostomy insertions [28]. The authors speculate that this effect is the result of post-insertion packing of the round window opening with a soft tissue graft, and subsequent fibrotic and neo-osteogenic changes leading to reduced RW membrane compliance. Other possible reasons for lower RHP rates in eRW insertions are extensive drilling of the so-called ‘hook-region’, with subsequent possible acoustic trauma, disruption of the vascular drainage and blockage of the endolymphatic system due to fibrous tissue formation [29].

Besides insertions via the extended round window- or cochleostomy approaches, SME insertions via so-called ‘pure round window approach’ and ‘round window enlargement’ have also been described, which do not require drilling the crista fenestrae [18, 21, 30]. Although in essence less traumatic than the other approaches, we believe the pure round window approach is only possible in patients with a favorable temporal bone anatomy and even in these cases, insertion of the plastic sheath that houses the electrode is often only possible after extensive drilling of the superior lip of the round window niche (round window enlargement) or carefully drilling away the crista fenestrae (extended round window). In our experience, and as also described by Shaul et al., insertion of the sheath in patients with an inadequately extended round window opening can lead to the deformation and compression of the sheath-opening [23]. Attempting to advance the array out of the compressed sheath-opening could lead to protrusion of the electrode through the side of the sheath. The silicone cochleostomy sizer tool which is supplied with the implant is, therefore, a valuable asset in determining whether an adequate access has been achieved, especially when the access to the scala tympani is challenging. Liebscher et al. described the scalar position and hearing outcomes of 156 patients implanted with the SME, 127 of whom via the round window, but did not elaborate on whether extensive drilling of the bony overhang preceded electrode insertion [21]. In the subset of patients for whom speech perception testing was performed, they were able to achieve a median speech recognition score of 75% (*n* = 148, range: 27.5–95%) using the Freiburg monosyllable word test. However, the fact that so little studies report on ‘pure round window’ insertions or do not exactly describe the procedure leading up to insertion of the electrode emphasizes the exceptionality of the anatomical circumstances necessary to insert the SME directly through the round window membrane. On the other hand, so-called ‘round window enlargement’, in which the posterosuperior lip of the bony overhang covering the round window niche is extensively drilled without additional intracochlear drilling, seems to deliver good results, as described by Ramos et al. and Iso-Mustajärvi et al., who reported on 18 SME insertions via a ‘round window membrane’ approach, but add to their description of surgical technique that the superior bony overhang of the round window niche was largely drilled away to achieve an optimal view of the round window membrane [20, 22]. Their technique may be comparable to what other authors describe as round window enlargement and resulted in residual hearing preservation (PTA_125-500_ < 80 dB) in 14/17 ears (82%).

In summary, each of the surgical techniques has its advantages and limitations. To achieve correct positioning of the SME with the highest chances of ST placement, the different round window techniques seem to be favored over the cochleostomy technique. Between the insertions through the round window, the pure round window approach would be the technique of choice if the individual anatomy and silicone sheath would allow it. However, in the majority of the cases insertion directly through the RW is not possible and for those cases, round window enlargement or round window extension will also provide excellent ST positioning and speech perception scores. On the other hand, the extended round window approach showed suboptimal results for preservation of residual hearing in this study, with a level of loss comparable to that of patients implanted via cochleostomy with translocation to the SV [4]. The best results specifically regarding residual hearing preservation seem to have been reported with the cochleostomy technique if the electrode does not translocate and is positioned entirely within the scala tympani [4, 22]. To accomplish this, one should focus on placing the cochleostomy as inferiorly as possible, to direct the sheath away from the spiral lamina in the direction of the floor of the scala tympani and minimize the risk of cochleostomy-associated translocation [31]. However, a great anatomical variation exists between individuals and, therefore, the risk of direct translocation to the SV should always be considered when using the cochleostomy approach.

The main limitation of the current study is that the number of participants is relatively small. The sample size was chosen to be able to compare to our previous study, in which we investigated CI outcomes using the SME implanted via cochleostomy. However, the direct comparison to this previous study was hampered by differences between the two patient populations, as the current study was conducted solely in participants with post-lingual hearing loss, with better preoperative pure tone audiometry thresholds and speech perception, and the surgery was conducted by three different surgeons. Additionally, residual hearing has been found to deteriorate beyond 24 months after surgery [32]. Therefore, the residual hearing loss observed in our study population may still proceed past the last follow-up timepoint. Lastly, the terminology used to describe the exact approach taken to access the scala tympani was found to differ in other studies, also complicating direct comparison; although several papers report on using the round window approach it is unclear whether this includes complete or partial removal of the bony overhang, or drilling of the crista fenestrae. Therefore, further studies investigating the effect of the different surgical approaches on CI outcome using this electrode are necessary.

## Conclusion

In this prospective study, we showed that correct positioning of the SME within the scala tympani can consistently be achieved using the extended round window approach, resulting in excellent levels of speech perception. However, the results also indicate that extending the round window opening can be suboptimal for the residual acoustic hearing, emphasizing that with this particular electrode there is no ‘all-purpose’ insertion approach for cochlear implantation. Where the anatomy is favorable, an insertion via the round window should be considered; if surgically feasible, patients *with* residual hearing should be implanted via ‘pure round window’ or ‘round window enlargement’ approach, whilst patients *without* residual hearing should be implanted via an extended round window approach. In cases where the anatomy is not favorable, i.e. in patients in whom round window insertions are not possible due to inadequate visibility of the RW membrane, an inferiorly placed cochleostomy may offer a solution and result in good levels of both residual hearing preservation and speech perception if the electrode is placed within the scala tympani.
